# Identification of Thyroid Receptor Ant/Agonists in Water Sources Using Mass Balance Analysis and Monte Carlo Simulation

**DOI:** 10.1371/journal.pone.0073883

**Published:** 2013-10-25

**Authors:** Wei Shi, Si Wei, Xin-xin Hu, Guan-jiu Hu, Cu-lan Chen, Xin-ru Wang, John P. Giesy, Hong-xia Yu

**Affiliations:** 1 State Key Laboratory of Pollution Control and Resource Reuse, School of the Environment, Nanjing University, Nanjing, People's Republic of China; 2 State Environmental Protection Key Laboratory of Monitoring and Analysis for Organic Pollutants in Surface Water, Jiangsu Provincial Environmental Monitoring Center, Nanjing, People's Republic of China; 3 Key Laboratory of Reproductive Medicine and Institute of Toxicology, Nanjing Medical University, Nanjing, People's Republic of China; 4 Department of Veterinary Biomedical Sciences and Toxicology Centre, University of Saskatchewan, Saskatoon, Saskatchewan, Canada; 5 Department of Zoology, and Center for Integrative Toxicology, Michigan State University, East Lansing, Michigan, United States of America; 6 Department of Biology and Chemistry and State Key Laboratory in Marine Pollution, City University of Hong Kong, Kowloon, Hong Kong, SAR, China; 7 School of Biological Sciences, University of Hong Kong, Hong Kong, SAR, China; Baylor College of Medicine, United States of America

## Abstract

Some synthetic chemicals, which have been shown to disrupt thyroid hormone (TH) function, have been detected in surface waters and people have the potential to be exposed through water-drinking. Here, the presence of thyroid-active chemicals and their toxic potential in drinking water sources in Yangtze River Delta were investigated by use of instrumental analysis combined with cell-based reporter gene assay. A novel approach was developed to use Monte Carlo simulation, for evaluation of the potential risks of measured concentrations of TH agonists and antagonists and to determine the major contributors to observed thyroid receptor (TR) antagonist potency. None of the extracts exhibited TR agonist potency, while 12 of 14 water samples exhibited TR antagonistic potency. The most probable observed antagonist equivalents ranged from 1.4 to 5.6 µg di-n-butyl phthalate (DNBP)/L, which posed potential risk in water sources. Based on Monte Carlo simulation related mass balance analysis, DNBP accounted for 64.4% for the entire observed antagonist toxic unit in water sources, while diisobutyl phthalate (DIBP), di-n-octyl phthalate (DNOP) and di-2-ethylhexyl phthalate (DEHP) also contributed. The most probable observed equivalent and most probable relative potency (REP) derived from Monte Carlo simulation is useful for potency comparison and responsible chemicals screening.

## Introduction

Increasing attention has been given to contaminants that can potentially interfere with the endocrine system [1]. Most research has especially been focused on androgen and estrogen homeostasis, and less information is available regarding disruption of the thyroid system [2]. Thyroid hormone (TH) is a key molecule involved in normal development of the brain of higher vertebrates and postembryonic development in lower vertebrates. Disruption of TH homeostasis during development of the central nervous system of children might cause neurological deficits and irreversible mental retardation [3]. Several synthetic chemicals from agriculture and industry, such as plasticizers and pesticides, exert toxic effects on the thyroid gland [4], [5].

Reporter gene-based whole-cell assays, which have high sensitivity and low background noise, have been widely used for biological and environmental screening to detect endocrine disrupting effects. The green monkey kidney fibroblast (CV-1) cell line based TH reporter gene assay is useful for predicting effects of some thyroid hormone disrupting chemicals and/or environmental samples [6]. In transient transformation assays, fold induction could be quite large. The transient transformation assay can also give more control over the specificity of response for agonists and antagonists.

Thyroid hormone disrupting effects have been detected in environmental samples including sediment extracts, indoor dust, industrial effluents and even water sources [7], [8]. Normal treatment processes in sewage treatment plants, including filtration, coagulation, aerobic biodegradation and ozonation are not considered to be effective for removal of endocrine disrupting chemicals [7]. Little information is available for thyroid hormone disrupting effects, although a range of thyroidal system inhibiting chemicals such as pesticides have previously been detected in the surface waters [9], [10].

To simplify data interpretation and facilitate quantitative risk assessment, the toxic potency of complex mixtures to modulate a defined biological response is often expressed relative to that of a well characterized standard chemical such as 5α-dihydrotestosterone (DHT) and flutamide for androgenic agonist and antagonist effect, or 17-β-estradiol [E2] for effects of estrogen agonists [11]. Relative potency (REP) related to the receptor mediated potencies is generally calculated as the ECi (i % relative inhibitory concentration) of a well characterized standard divided by the ECi of a sample or the tested chemicals, such that the ratio describes the potency of the sample relative to the standard [12]. Relative potencies based on such a ratio of point estimates are valid only under limited conditions [13]. It is assumed that dose-response relationships for chemicals and the well characterized standard are parallel. As a result, the REP of one chemical or sample at different inhibitory concentrations exhibit equal efficacy. However, there is little reason to assume that dose–response relationships for the complex mixtures analyzed by the same in vitro bioassay will be parallel or exhibit equal efficacy [14]. For nonparallel dose–response relationships, the REP is a function of dose or concentration factor. The relationship described at single levels of response, such as the EC_20_, EC_50_ and EC_80_ are not constant over the entire range of responses [15]. As a result the REP ranges were recognized to identify the effects of environmental samples and tested chemicals to provide a quantitative estimate of relative potency that is valuable for comparing among samples [16]. Because REP ranges describe the uncertainty due to nonparallel slopes, the standard range has been defined as 20 to 80% (REP_20–80_ range) of the maximum response achieved for the standard compound (20–80% std. max.). The REP_20–80_ ranges are valuable for comparing samples, however, they are not sufficient for mass balance analysis considering about the uncertainty induced. Moreover, the distributions of the REP values and the most probable values were also ignored. Monte Carlo simulation can be employed to further accommodate the uncertainties and quantify variability [17]. Monte Carlo simulation is the most commonly used method to quantify variability to accommodate the uncertainties in risk assessments recommended by the National Academy of Sciences and USEPA [18], [19]. Through repetitive calculations, a probability density function or cumulative density function for the output can be generated [20], [21]. This technique could provide a quantitative way to estimate the probability distributions for REPi within the validity of the assessment model. Moreover, the most probable values can be estimated.

The three objectives of the study were to: 1) Build an assessment technique based on Monte Carlo simulation to determine REP ranges and chemicals responsible for the observed potency by use of mass balance analysis; 2) Evaluate the TR antagonist potencies in waters in the Yangtze River Delta; 3) Identify the compounds responsible for thyroid-active potency of extracts using Monte Carlo simulation based mass balance analysis.

## Materials and Methods

### Study locations

Yangtze River, Tai Lake, Huai River and groundwater are sources of drinking water in Yangtze River Delta region, which include more than 30 water sources. The Yangtze River is the most important source of drinking water to more than 50 million residents in 8 large cities in the Yangtze River Delta [22], [23]. Tai Lake serves 3% of the population in China as an indispensable source of drinking water, agriculture, aquaculture and industrial plants [24], [25]. The Huai River is a densely populated and industrialized urban area in Chain, and serves more than 50 million people as source of drinking water [26], [27]. Moreover, more than 20% of the Yangtze River Delta population depends on ground water for drinking water from either a public source or private wells. The drinking water treatment processes including coagulation, sedimentation, filtration and chlorination are commonly used. The advanced treatment processes, such as activated carbon adsorption, ultra filtration membrane and magnetic ion exchange resin, have also been adapted in these waterworks.

### Chemicals

According to our previous studies, phthalates and pyrethroids which exhibited strong thyroid hormone disrupting activities were selected [6], [28]. The information of the chosen chemicals was given in Table 1. All phthalate standards were 99.5% pure, and pyrethroid standards were 99% pure. Internal standards, di-n-butyl phthalate-d4, bis(2-ethylhexyl)Phthalate-3,4,5,6-d4 and ^13^C-PCB 141 were purchased from Sigma Chemical Co. (St. Louis, MO, USA). Chemicals for bioassays including _L_-3,5,3′-triiodothyronine (T_3_) and 3-(4,5-dimethylthiazol-2-ol)-2,5-diphenyltetrasodium bromide tetrazolium (MTT) with the purity of over 97% were obtained from Sigma Chemical Co. (St. Louis, MO, USA). The plasmid phRL-tk (used as internal control for transfection efficiency), containing Renilla luciferase gene, was purchased from Promega (Promega, Madison, WI, USA). No specific permissions were required for these locations/activities and the field study did not involve endangered or protected species.

**Table 1:** Sources, formula and CAS of tested chemicals.

Classes (Providers)	Name	CAS	Formula
Plasticizers (Labor Dr.Ehrenstorfer-Schafers, Germany)	Dibutyl phthalate (DNBP)	84-74-2	C_16_H_22_O_4_
	di-2-ethylhexyl phthalate (DEHP)	117-81-7	C_24_H_38_O_4_
	dimethyl phthalate (DMP)	131-11-3	C_10_H_10_O_4_
	diethyl phthalate (DEP)	84-66-2	C_12_H_14_O_4_
	benzyl butyl phthalate (BBP)	85-68-7	C_19_H_20_O_4_
	bis(2-ethylhexyl) adipate (DEHA)	103-23-1	C_22_H_42_O_4_
	diisobutyl phthalate (DIBP)	84-69-5	C_16_H_22_O_4_
	di-n-octyl phthalate (DNOP)	117-84-0	C_24_H_38_O_4_
Pyrethroid Insecticides (Sigma Chemical Co., St. Louis, MO, USA)	Cyfluthrin	68359-37-5	C_22_H_18_C_l2_FNO_3_
	Cypermethrin	52315-07-8	C_22_H_19_C_l2_NO_3_
	Deltamethrin	52918-63-5	C_22_H_19_Br_2_NO_3_
	Dichloran	99-30-9	C_6_H_4_ Cl_2_ N_2_ O_2_
	Fenvalerate	51630-58-1	C_25_H_22_CINO_3_
	L-Cyhalothrin	91465-08-6	C_23_H_19_ClF_3_NO_3_
	Pendimethalin	40487-42-1	C_13_H_19_N_3_O_4_
	Permethrin	52645-53-1	C_21_H_20_C_l2_O_3_
	Tefluthrin	79538-32-2	C_17_H_14_ClF_7_O_2_
	Tetrachlorvinphos	7696-12-0	C_19_H_25_NO_4_
	Cycloprothrin	63935-38-6	C_26_H_21_C_l2_NO_4_
	Cyhalothrin	91465-08-6	C_23_H_19_ClF_3_NO_3_
	Etofenprox	80844-07-1	C_25_H_28_O_3_

### Sample preparation and analysis

**Figure 1:**
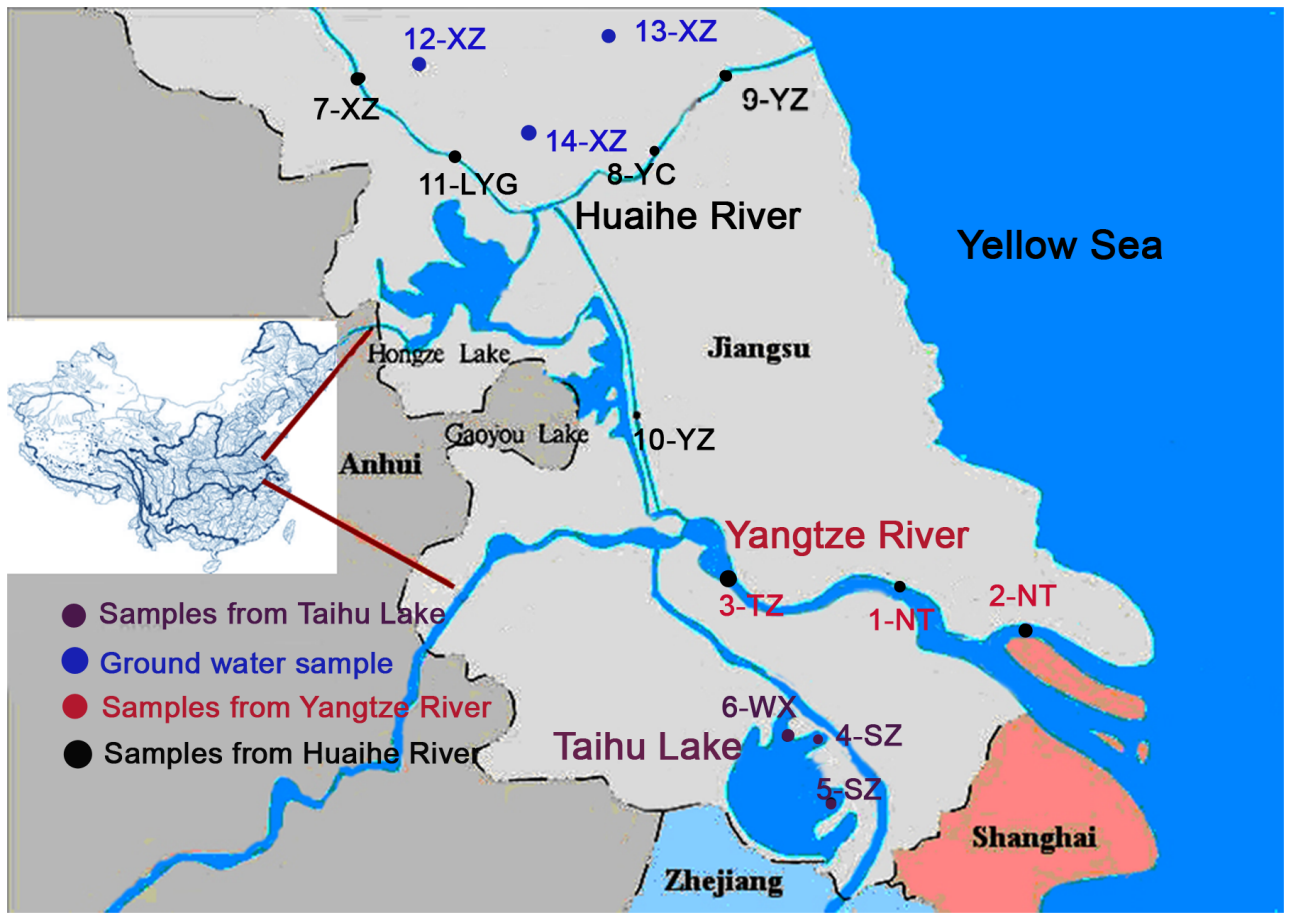
Map of the chosen water sources from Yangtze River, Taihu Lake, Huaihe River and groundwater.

Fourteen water samples were collected from locations in the Yangtze River Delta in May 2009 (Figure 1). Sampling locations were from the Yangtze River (1-NT, 2-NT and 3-TZ), Tai Lake (4-SZ, 5-SZ and 6-WX), Huai River (7-XZ, 8-YC, 9-YC, 10-YZ and 11-LYG), and ground water (12-XZ, 13-XZ and 14-XZ). A water sample of 15 L (10 L for bioassay and 5 L for chemical analysis) were collected at each location. The water samples were collect in water sources near the intake pipe of the drinking water treatment plants.

Samples and procedure blanks (Milli-Q water) were extracted by use of a modification of a previously published protocol [29], [30]. Solid phase extraction (SPE, 500 mg Oasis HLB cartridges, Waters, USA) was performed. Cartridges were activated and conditioned with high-purity hexane (Merck), dichloromethane (TEDIA), acetone (TEDIA) and methanol (TEDIA) sequentially. Absorbed compounds were eluted stepwise with 10 mL hexane, 10 mL hexane: dichloromethane (4∶1), followed by 10 mL acetone: methanol (1∶1). Extracts for bioassays were reconstituted in DMSO and were diluted 12.5, 25, 50, 100, and 200 times the original concentration in source water.

Plasticizers were quantified using a Thermo TSQ Quantum Discovery triple-quadrupole mass spectrometer (San Jose, CA, USA) in multiple-reaction monitoring (SRM) mode. The precision of the method was quantified by relative standard deviation (SD), and was determined by replicate extractions (n = 3) of a single sample.

### Cell culture and transfection

A green monkey kidney fibroblast (CV-1) cell line lacking functional TR was obtained from the Institute of Biochemistry and Cell Biology in Shanghai, Chinese Academy of Science [31]. The cell line was cultured in Dulbecco's modified Eagle's medium (DMEM) (Sigma, St. Louis, MO, USA) supplemented with 10% fetal bovine serum (FBS; Gibco, Invitrogen Corporation, Carlsbad, CA, USA), 100 U/mL penicillin (Sigma) and 100 µg/mL streptomycin (Sigma) in an atmosphere containing 5% CO_2_ at 37°C. Cells were seeded in 48-well microplates at a density of 5.0×10^4^ cells per well. Twelve hours later, cells were transfected with 0.25 µg Gal4 responsive luciferase reporter pUAS-tkluc, 0.1 µg pGal4-L-TR using 2.5 µg Sofast TM transfection reagent per well. The transfection efficiency of the transfection was 65±4%. After further 12 h incubation, cells were exposed to various concentrations of standard compounds and sample extracts dissolved in medium for 24 h. Our previous study has indicated that the relative potency for inducing luciferase activity of natural thyroid hormone T3 is greater than T4 [32]. Moreover T4 is prone to be metabolized and T3-dependent activation was chosen to study thyroid hormone disrupting potencies in this study (see Figure S1 in File S1) [33].

### Toxic equivalents estimation for antagonist potencies

The TR agonistic and antagonistic equivalents were calculated to measure the TH disrupting potencies and assess potential health risks of the tested waters. The dose-response curves of tested samples and chemicals were fitted to experimental data by use of a three parameter sigmoid model (Eq. 1):
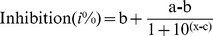
(1)where a, b and c are curve-fitting parameters of the model and x is the concentration.

For antagonist potencies, all the effect are compared with the control level of the agonist and this level should be comparable in analogous biochemical processes. For example, 5.0×10^−9^ mol T_3_/L were always employed as control in the ant-thyroid hormone effect detecting assays and this level could also be defined as std. max (100%). Di-n-butyl phthalate (DNBP) was chosen as the well characterized standard for the TR antagonist potency in this study [34].

The observed equivalent (ObTH-EQ) for environmental samples were derived from the concentration factor at which the EC_i_ (i% relative inhibitory concentration, inhibition by i%) of sample was obtained based on the following equation and reflected the bioassay results (Eq 2).
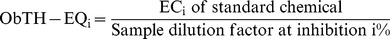
(2)


In most cases, the dose-response relationships for sample extracts or model chemical are not parallel, and the ObTH-EQi is not a single point but a range (Eq. 3). The range is calculated based on Monte Carlo simulation using the algorithm @ Risk (5.7 version, Palisade Corporation, Newfield, NY, USA). At the same time, the maximum, minimum, mean value and distribution of ObTH-EQi were obtained. ObTH-EQi is the equivalent determined at a defined probability level as Yi. The ObTH-EQpro is defined as the ObTH-EQ with the highest probability level.
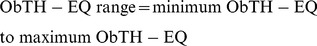
(3)


The relative potency (REP) for chemicals could be derived from the concentrations at which the EC_i_ (i% relative inhibitory concentration, inhibition by i%) of sample was obtained based on the following equation and reflected the bioassay results (Eq. 4).
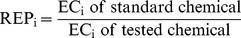
(4)


In most cases, dose-response relationships for different chemicals are not parallel, and the REPi is also not a single point. This REPi of a chemical could be arbitrarily defined as the REP_20_ or REP_50_ as well as a range which is not sufficient for mass balance analysis. The most appropriate REPi was defined as a function of concentration.

Predicted EQs represented estimates of sample toxicity based on measured chemical concentrations in environmental samples based on equation 4. Concentration addition was used for mixture toxicity estimation. Predicted EQ (PrTH-EQi) was calculated by multiplying the contaminant concentration in water (c) by the most probable REP (REP-pro) values of the related chemical detected based on Monte Carlo simulation.
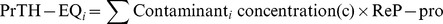
(5)


### Data analysis

Values were reported as mean ± SD (n = 3). In the bioassays triplicate wells were done for each treatment. For agonistic potencies, treatments were compared to the vehicle control; while for antagonistic potencies, treatments were compared to 5.0×10^−9^ mol/L T_3_ positive control groups respectively. Data were analyzed by use of one-way ANOVA, followed by Duncan's multiple comparisons test when appropriate using SPSS statistical software (version 11, SPSS Inc., Chicago, Illinois). Curve-fitting analysis was carried out with GraphPad 5.4 (San Diego, CA, USA). The level of significance was set at ** p<0.01. To accommodate the uncertainties associated within the REP and equivalent calculation process due to the use of point concentration data, Monte Carlo simulation technique was applied in this study. The fitted distribution functions of the inhibition data were used as simulated data sets. The next, 10000 Monte Carlo iterations were performed based on the @Risk software (5.7 version, Palisade Corporation, Newfield, NY, USA). A probabilistic distribution of REP for standard and equivalent for mixture instead of the point values, were then generated as calculation results.

## Results and Discussion

### Cell viability and system creditability

None of the individual chemicals or extracts affected viability or proliferation of CV-1 cells alone or in the presence of 5.0×10^−9^ mol T_3_/L. No cytotoxic effects of solvent or water extracts were observed by microscopic examination throughout the transfection assay. The natural TR ligand T_3_ induced luciferase activity in a concentration-dependent manner in the CV-1 reporter assay ranging from 1.0×10^−10^ mol/L to 1.0×10^−6^ mol/L. No significant induction of luciferase was observed in any of the solvent controls (data not shown). Recoveries of chemicals in instrumental analysis were between 82% and 110% (see Table S1 in File S1).

### TR ant/agonist potency

**Figure 2:**
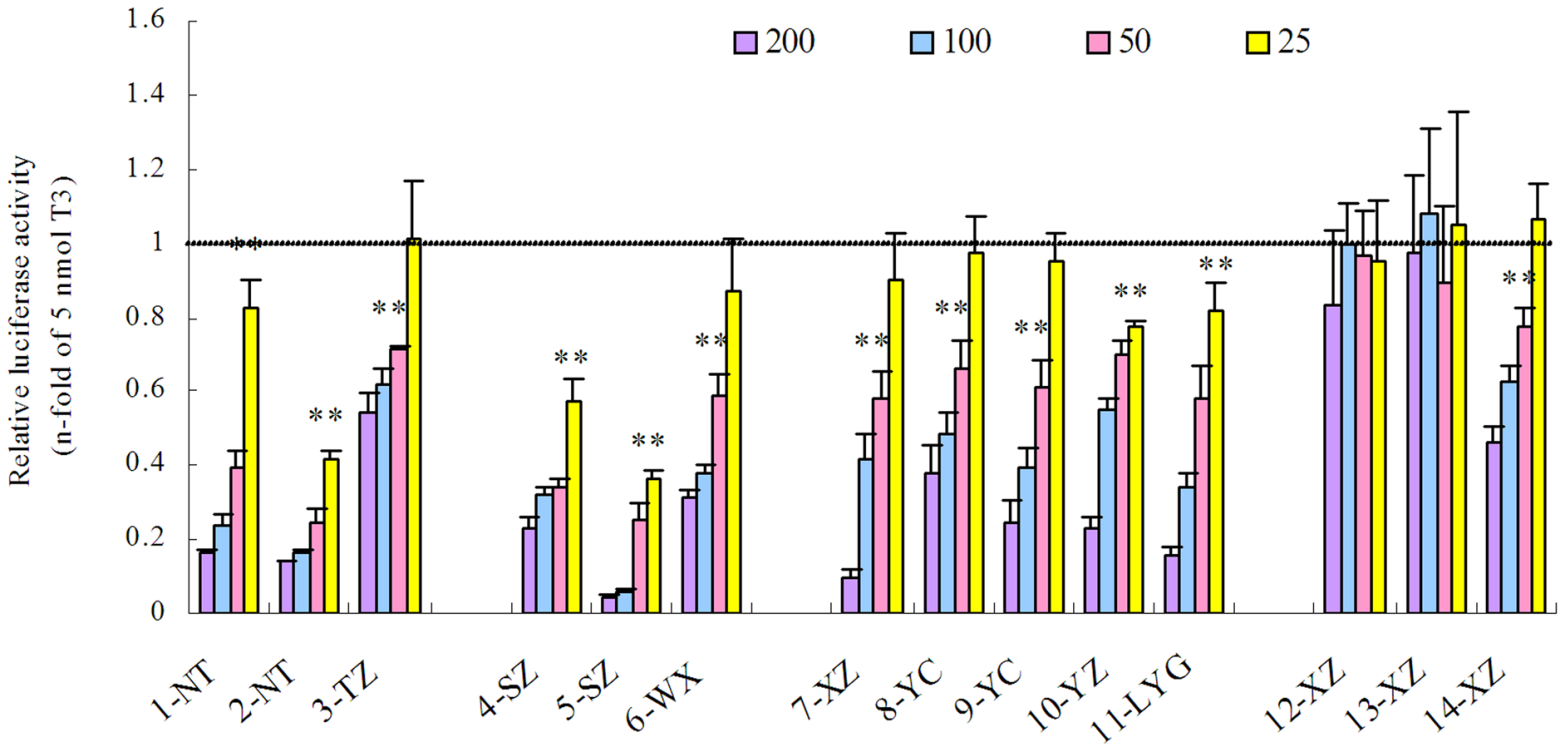
Concentration-dependent TR antagonist activities in the water extracts measured by the CV-1 cell line TR reporter gene assay. Water extracts were tested at 25, 50, 100 and 200 times the original concentration. Cells were exposed to extracts in parallel with 5_3_ as indicated by the dashed line. The TR antagonist activity was expressed as relative expression versus the untreated cells with 5 nmol T_3_ (control) (mean ± SD). Significant differences were indicated by asterisks (** p<0.01). The results of statistical analysis at higher concentrations which also exhibited significant differences (**p<0.01) were not shown.

None of the tested extracts displayed any TR agonistic potency. However, twelve of the fourteen tested source water extracts exhibited TR antagonistic potencies in a concentration-dependent manner (Figure 2). All the water extracts from Tai Lake, the Yangtze River and Huai River displayed TR antagonist potency. The two extracts which exhibited no antagonist potency were both from ground water. Extracts of all the surface water with the maximal concentration factors (200 times the original concentration in the source water) decreased luciferase expressions to less than 40% of 5 nmol/L T_3_ potency, respectively.

### Concentrations of TR antagonist

Individual concentrations of phthalate esters, and pyrethroid insecticides in source water were shown (Table 2). Phthalate esters were detectable in all samples analyzed except DNOP at location 1-NT. Concentrations of DNBP, DIBP and DEHP were the highest, ranging from 2.1×10^1^ to 3.2×10^3^ ng/L, 1.9×10^2^ to 1.2×10^4^ ng/L and 4.0×10^1^ to 2.1×10^3^ ng/L, respectively. Pyrethroid insecticides were detected in none of the samples. Great concentrations of DIBP were also detected. This is in accordance with previous studies regarding the concentrations of phthalate esters in water, soil and dust sample in China [35], [36], [37], [38]. The results indicated the common use of DIBP in the Yangtze River Delta. Concentrations of DEHP were less than DNBP or DIBP, which is consistent with the results of previous studies in China [35]. Concentrations of DNBP, DEHA, DEP and deltamethrin in waters were less than the national standard for tap water in China, which are 8, 300 and 20 µg/L, respectively [39]. However, there is no standard for concentrations of DIBP in source water. Concentrations of phthalate esters in Tai Lake were greater than those in most of the other samples. This result indicated that in more urbanized areas, infiltration of treated or untreated sewage maybe the source of pollution in Tai Lake. Phthalate esters were also detectable in all three ground water, with the greatest concentration found in 14-XZ, and thus may potentially serve as a precautionary indicator of ground water contamination.

**Table 2:** Concentrations of thyroid hormone disrupting compounds (ng/L) in water sources.

Chemicals	Locations													
	1-NT	2-NT	3-TZ	4-SZ	5-SZ	6-WX	7-XZ	8-YC	9-YC	10-YZ	11-LYG	12-XZ	13-XZ	14-XZ
DMP	2.7×10^0^	4.1×10^1^	2.0×10^2^	7.4×10^0^	8.0×10^1^	3.1×10^1^	7.5×10^1^	1.9×10^1^	4.8×10^1^	2.8×10^1^	2.4×10^1^	5.4×10^1^	7.0×10^1^	1.9×10^1^
DEP	3.8×10^1^	4.2×10^1^	3.7×10^2^	1.1×10	1.2×10^2^	4.5×10^1^	9.0×10^1^	2.9×10^1^	7.4×10^1^	4.1×10^1^	1.2×10^2^	3.7×10^0^	7.5×10^1^	1.9×10^1^
DIBP	1.9×10^3^	1.2×10^4^	1.9×10^2^	3.6×10^3^	4.4×10^3^	1.7×10^3^	2.1×10^3^	1.8×10^3^	2.1×10^3^	2.1×10^3^	3.2×10^3^	1.9×10^2^	3.0×10^2^	2.6×10^3^
DNBP	1.3×10^3^	1.4×10^3^	2.1×10^2^	2.1×10^3^	1.7×10^3^	1.1×10^3^	1.4×10^3^	9.0×10^2^	1.2×10^3^	1.4×10^3^	3.2×10^3^	1.4×10^2^	2.0×10^1^	1.3×10^3^
BBP	1.7×10^2^	3.1×10^2^	9.4×10^1^	1.1×10^1^	1.5×10^1^	1.4×10^0^	4.5×10^1^	3.5×10^1^	5.3×10^2^	2.9×10^1^	4.7×10^1^	1.3×10^1^	2.5×10^1^	1.1×10^1^
DEHA	2.0×10^2^	3.3×10^1^	4.0×10^2^	2.8×10^−1^	2.2×10^0^	5.4×10^3^	7.2×10^2^	5.4×10^0^	2.3×10^2^	2.2×10^1^	5.9×10^2^	1.0×10^0^	3.8×10^0^	7.2×10^0^
DEHP	9.2×10^1^	1.8×10^3^	2.1×10^3^	5.5×10^1^	7.3×10^2^	4.5×10^1^	8.6×10^1^	1.3×10^2^	4.5×10^2^	1.7×10^2^	2.8×10^2^	4.0×10^1^	1.6×10^2^	1.8×10^2^
DNOP	N.D	1.0×10^2^	3.6×10^3^	1.2×10^1^	1.7×10^1^	2.2×10^2^	2.7×10^2^	3.0×10^1^	5.7×10^2^	6.4×10^1^	5.4×10^2^	2.1×10^1^	2.8×10^2^	2.7×10^1^
ΣPyrethroid Insecticides	N.D	N.D	N.D	N.D	N.D	N.D	N.D	N.D	N.D	N.D	N.D	N.D	N.D	N.D

N.D: not detectable, <LOD.

### Toxic equivalents

**Figure 3:**
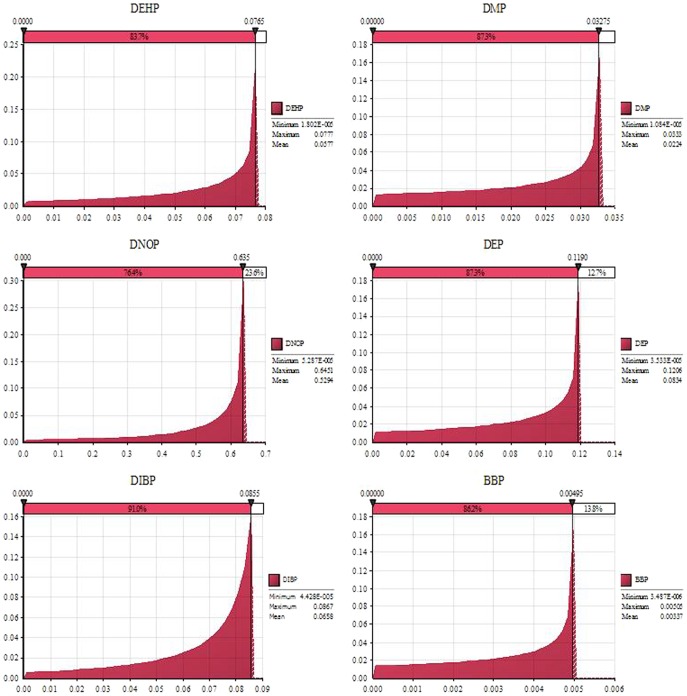
The probability density distributions of relative potency (REP) for the detected phthalate esters (μmol tested chemical·L^−1^/µmol DNBP·L^−1^).

**Figure 4:**
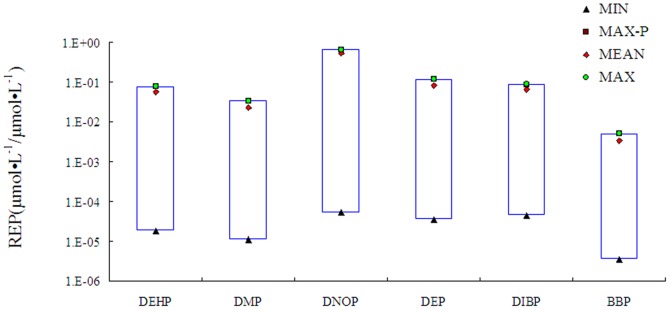
Relative potency (REP) ranges for the detected phthalate esters (µmol tested chemical·L^−1^/μmol DNBP·L^−1^).

TR antagonistic potencies of detected phthalate esters were evaluated by use of reporter gene assay and the dose-response curves were fitted to these experimental data by use of the three-parameter, sigmoid model (see Table S2 in File S1). All phthalate esters except DEHA exhibited measurable TR antagonistic potencies. The probability density distributions of REP (µmol tested chemical·L^−1^/µmol DNBP·L^−1^) for phthalate esters were developed based on Monte Carlo simulation (Figure 3). Most of the values distributed closely to the maximal values. The maximal REP (MAX), mean REP (MEAN) and the most probable REP (REP-pro) were similar. The REP ranges (µmol tested chemical·L^−1^/µmol DNBP·L^−1^) for the detected chemicals are shown in Figure 4. DNOP exhibited the greatest REP ranges while BBP exhibited the least REP ranges. The REP values for the other phthalate esters were similar. The dose-response curves for REP and inhibition of the detected chemicals are shown in Figure S2 in File S1, and totally different shapes of curves were observed for these phthalate esters. According to these dose-response curves inhibition ranges for the REP-pro were obtained (see Table S2 in File S1). The result indicated that the most probable inhibition ranges were from 75% to 93%, but not 50%. However, EC_50_ has always been employed in previous studies to determine REPs of chemicals, which might have resulted in underestimation or overestimation because of non-parallelism of the dose-response curves [16]. Inhibition ranges were quite different for the various chemicals and REP-pro was employed to evaluate the effect of the related chemical. Previous studies have indicated that the REP ranges are not suitable for mass balance analysis, because the range is not a settled number. In this study, the REP-pro which was defined as the most probable REP was used to predict the PrTH-EQ of the samples at the greatest probability (Table 3). The REP-pro derived PrTH-EQ (PrTH-EQ-pro) for the water sources ranged from 1.8×10^−1^ to 4.0 µg/DNBP L, with the greatest concentration of TH equivalents found at location 11-LYG from the Huai River (Table 3).

**Table 3:** The most probable observed equivalent (ObTH-EQ–pro) derived from reporter gene assays and the most probable predicted equivalent (PrTH-EQ-pro) derived from instrumental analysis (µg/DNBP L).

	ObTH-EQ–pro	PrTH-EQ-pro
1-NT	2.2×10^0^	1.4×10^0^
2-NT	2.8×10^0^	2.6×10^0^
3-TZ	1.4×10^0^	8.2×10^−1^
4-SZ	2.8×10^0^	2.4×10^0^
5-SZ	2.8×10^0^	2.2×10^0^
6-WX	2.2×10^0^	1.4×10^0^
7-XZ	8.3×10^−1^	1.9×10^0^
8-YC	1.4×10^0^	1.1×10^0^
9-YC	1.4×10^0^	2.0×10^0^
10-YZ	8.3×10^−1^	1.7×10^0^
11-LYG	5.6×10^0^	4.0×10^0^
12-XZ	N.E	1.8×10^−1^
13-XZ	N.E	3.2×10^−1^
14-XZ	2.8×10^0^	1.5×10^0^

N.E: No effect was observed.

The probability density distributions of ObTH-EQs were developed based on Monte Carlo simulation (see Figure S3 in File S1). The ObTH-EQs of the detected water sources were equivalent. The minimum values ranged from 6.99×10^−12^ to 2.58×10^−10^ µmol DNBP/L. The maximal values (MIN) ranged from 5.13×10^−6^ to 2.95×10^−5^ µmol DNBP/L. The mean values were similar to or equivalent to the most probable values (see Figure S4 in File S1), with the greatest values occurring at location 11-LYG. The most probable ObTH-EQs (ObTH-EQs–pro) for the water sources ranged from 8.3×10^−1^ to 5.6 µg DNBP/L (Table 3).

**Figure 5:**
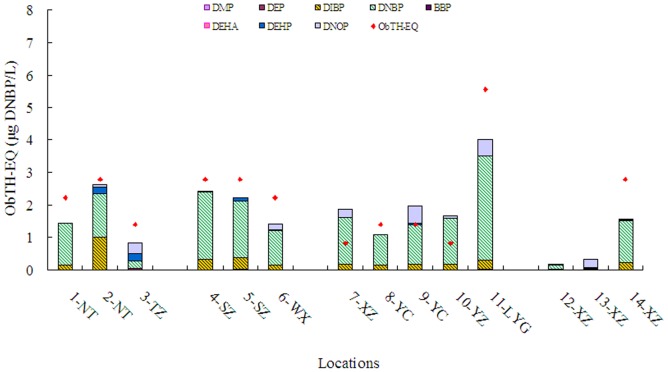
Contributions of individual chemicals to the ObTH-EQs in water samples.

When the amounts of ObTH-EQs measured by the bioassay were compared with those calculated from the concentrations of detected TR antagonists (PrTH-EQ) based on the REP-pro, more than 55.8% of the total ObTH-EQs in water sources were contributed by detected phthalate esters (Figure 5). With the greatest concentration and greatest TR antagonist potency, DNBP accounted for most of the ObTH-EQs in most locations. DIBP, DNOP and DEHP accounted for 1.2% to 35.9%, 0 to 36.5%, 0.4% to 16.2% of the ObTH-EQs, respectively. Contributions of DIBP and DNOP were relatively great, a result that has not been reported previously. This can be attributed to use of DIBP and DNOP in the Yangtze River Delta, which is consistent with results of previous studies conducted in Guangdong, China [35]. DNBP is the major TR antagonist potency in water sources at all the detected sites, while DIBP, DNOP and DEHP also contributed. Standards established in July, 2012 for drinking water quality have added 71 monitoring objectives. However, DIBP and DNOP were not included. Less than 50% of the potencies were contributed by the detected chemicals for sample from Yangtze River. This indicated that unknown chemicals contributed most of the TR antagonist potency in groundwater. These thyroid-disrupting chemicals in source water could be a major concern if drinking water treatment does not remove these compounds. Previous studies have indicated that phthalate could be efficiently removed by macroporous OH-type strongly basic anion exchange resin [40] and modified activated carbon [41]. However limited information is available for the removal efficiency of other advanced treatment processes combined with the commonly used process for more thyroid-disrupting chemicals. Further study is needed to evaluate the associated removal efficiency in water works, which could be benefit the risk assessment for human health.

Dose-response curves for complex mixtures and standard chemicals analyzed by *in vitro* bioassay will not be parallel or exhibit equal efficacy to the standard [42], [43]. As a result, REP ranges including 20% inhibition, 50% inhibition and 80% inhibition were recommended for toxicity identification. However, REP ranges have been considered unsuitable for mass balance analysis. Choice of an appropriate model to describe a concentration–response curve is often arbitrary, so when the estimation technique involves extrapolation beyond the range of observed data the results could be misleading. In such cases, REP ranges, especially the 50% inhibition and 80% inhibition will not be estimated exactly. In this study, a new approach was developed based on Monte Carlo simulation for the estimation of REP and equivalent for standard and mixture for the very first time. Using this approach, REP values for the standard and the equivalent values for the mixture can be calculated rapidly for thousands of points along the standardized range of response, and the maximum, mean, minimum and most probable values could be generated. The most probable observed equivalent concentration derived from reporter gene assays and the most probable REP for detected chemicals could further be combined for the mass balance analysis. This method could provide REP ranges and equivalent ranges for comparison of risk potential posed by different standard or mixture and also for responsible chemicals searching. This could avoid inaccurate estimates of risk, due to the limitation of the relative studies. The most probable observed equivalent and most probable REP derived from Monte Carlo simulation are not only suitable for comparative purposes but also improve the accuracy of mass balance analysis.

## Conclusion

In conclusion, a novel approach was developed based on Monte Carlo simulation for the estimation of relative potency (REP) and thyroid hormone (TH) equivalents for standard compounds and mixtures. Using this approach, REP values for the model compound standards and concentrations of equivalents for mixtures can be rapidly calculated and thousands of points along the standardized range of response, and the maximum, mean, minimum and most probable values were generated. The most probable observed equivalent derived from reporter gene assays and the most probable REP for detected chemicals could further be combined for use in a mass balance analysis. This method could provide REP ranges and equivalent ranges for comparison of risk potential posed by different standard or mixture and also for responsible chemicals searching. Using this approach the presence of thyroid active chemicals and their toxicity potential in the most commonly used drinking water sources in the Yangtze River Delta was investigated. None of the water extract exhibited thyroid receptor (TR) agonist activity, but the occurrences of strong antagonist activities were more common in all the commonly used water sources except ground water. DNBP, DIBP, DNOP and DEHP contributed most for the observed antagonist toxic unit. More attention should be paid to these chemicals for the further management consideration. The most probable observed equivalent and most probable REP derived from Monte Carlo simulation are not only suitable for comparative purposes but also the estimate for risk assessment or mass balance analysis.

## Supporting Information

File S1 **Supporting information.**
(DOC)
