# Supplementary material for: Identification of Thyroid Receptor Ant/Agonists in Water Sources Using Mass Balance Analysis and Monte Carlo Simulation
Source: PLoS One. 2013 Oct 25;8(10):e73883. doi: 10.1371/journal.pone.0073883 (PMC3808374; doi:10.1371/journal.pone.0073883)
Supplement: File S1 — Supporting information. (DOC) [file pone.0073883.s001.doc]

**Identification of thyroid receptor ant/agonists in water sources using mass balance analysis and Monte Carlo simulation**

Wei Shi^1^, Si Wei^1^, Xin-xin Hu^1^, Guan-jiu Hu^2^, Cu-lan Chen^2^, Xin-ru Wang^3^,

John P. Giesy^1, 4, 5, 6, 7^, Hong-xia Yu^1*^

^1^ State Key Laboratory of Pollution Control and Resource Reuse, School of the Environment, Nanjing University, Nanjing, People’s Republic of China

^2^ State Environmental Protection Key Laboratory of Monitoring and Analysis for Organic Pollutants in Surface Water, Jiangsu Provincial Environmental Monitoring Center, Nanjing, People’s Republic of China

^3^ Key Laboratory of Reproductive Medicine & Institute of Toxicology, Nanjing Medical University, Nanjing, People’s Republic of China

^4^ Department of Veterinary Biomedical Sciences and Toxicology Centre, University of Saskatchewan, Saskatoon, Saskatchewan, Canada

^5^ Department of Zoology, and Center for Integrative Toxicology, Michigan State University, East Lansing, MI, USA

^6^ Department of Biology & Chemistry and State Key Laboratory in Marine Pollution, City University of Hong Kong, Kowloon, Hong Kong, SAR, China

^7^ School of Biological Sciences, University of Hong Kong, Hong Kong, SAR, China

Address correspondence to H. Yu.

Prof. Hongxia Yu, PhD: School of the Environment, Nanjing University, Nanjing, 210093, China. Tel.: +86 25 8968 0356, Fax: +86 25 8968 0356, E-mail: yuhx@nju.edu.cn

**Supporting Information, Table S1.** The recovery, limit of quantification (LOQ) and limit of detection (LOD) of the chemicals for instrumental analysis.

|  | Chemicals | Recovery (%) | RSD (%) | LOD (ng/L) | LOQ (ng/L) |
| --- | --- | --- | --- | --- | --- |
| Plasticizers | DMP | 94 | 3.3 | 4.3×10^-1^ | 1.3×10^0^ |
|  | DEP | 84 | 1.3 | 4.3×10^-1^ | 1.3×10^0^ |
|  | DIBP | 89 | 6.7 | 1.0×10^-1^ | 3.0×10^-1^ |
|  | DNBP | 97 | 4.2 | 6.7×10^-1^ | 2.0×10^0^ |
|  | BBP | 89 | 3.2 | 4.3×10^-1^ | 1.3×10^0^ |
|  | DEHA | 110 | 5.4 | 3.3×10^-1^ | 1.0×10^0^ |
|  | DEHP | 94 | 9.6 | 1.0×10^-1^ | 3.0×10^-1^ |
|  | DNOP | 89 | 3.7 | 2.3×10^-1^ | 7.0×10^-1^ |
| Pyrethroid pesticides | Dichloran | 88 | 8.8 | 5.0×10^-2^ | 1.8×10^-1^ |
|  | Tefluthrin | 90 | 3.6 | 6.0×10^-2^ | 1.9×10^-1^ |
|  | Pendimethalin | 91 | 1.3 | 6.0×10^-2^ | 1.9×10^-1^ |
|  | Tetrachlorvinphos | 95 | 2.0 | 2.5×10^-1^ | 8.2×10^-1^ |
|  | L-Cyhalothrin | 90 | 3.0 | 2.0×10^-2^ | 6.0×10^-2^ |
|  | Permethrin1 | 82 | 3.6 | 2.0×10^-2^ | 6.0×10^-2^ |
|  | Permethrin2 | 93 | 1.3 | 1.1×10^-1^ | 3.7×10^-1^ |
|  | Cyfluthrin isomers | 89 | 6.3 | 7.0×10^-2^ | 2.3×10^-1^ |
|  | Cypermethrin isomers | 88 | 6.9 | 7.0×10^-2^ | 2.2×10^-1^ |
|  | Fenvalerate1 | 93 | 2.7 | 4.0×10^-2^ | 1.3×10^-1^ |
|  | Fenvalerate2 | 84 | 3.6 | 4.0×10^-2^ | 1.4×10^-1^ |
|  | Deltamethrin | 93 | 6.5 | 4.0×10^-2^ | 1.3×10^-1^ |
|  | Cycloprothrin | 87 | 2.4 | 2.0×10^-2^ | 6.0×10^-2^ |
|  | Cyhalothrin | 91 | 3.1 | 2.0×10^-2^ | 6.0×10^-2^ |
|  | Etofenprox | 86 | 2.2 | 2.0×10^-2^ | 6.0×10^-2^ |

LODs: Limits of detection (S/N =3); LOQs: Limits of quantitation (S/N =10); Concentrations are expressed as mean ± SD (n=3)

**Supporting Information, Table S2.** The parameter for dose-response curves, the most probable REP (REP-pro) (μmol tested chemical·L^-1^/μmol DNBP·L^-1^)and the inhibition ranges of the phthalate esters.

| Chemicals | a | b | c | REP-pro (probability) | inhibition ranges |
| --- | --- | --- | --- | --- | --- |
| DMP | 1.013 | 0.8561 | -5.437 | 0.033 (15.5%) | 90.8%-91.6% |
| DEP | 0.9991 | 0.7972 | -5.61 | 0.12 (23.5%) | 89.9%-91.6% |
| DIBP | 1.007 | 0.8811 | -4.792 | 0.086 (7.4%) | 88.2%-88.8% |
| DNBP | 0.9562 | 0.4653 | -5.656 | 1 | / |
| DNOP | 0.9993 | 0.5228 | -5.646 | 0.64 (21.2%) | 74.9%-79.9% |
| BBP | 1.006 | 0.8815 | -4.797 | 0.005 (33.0%) | 91.3%-92.6% |
| DEHP | 0.9987 | 0.7058 | -5.112 | 0.077 (11.0%) | 85.0%-87.4% |

**Supporting Information, Figure S1.** Concentration-dependent luciferase activities in CV-1 cell line TR reporter gene assay treated with T_3_. Results are expressed as mean ± SD (n = 3).

**Supporting Information, Figure S2.** The dose-response curves for relative potency (REP, μmol•L^-1^/μmol•L^-1^) and inhibition of the detected chemicals (μg DNBP/L).

**Supporting Information, Figure S3.** Probability density distributions of observed equivalent (ObTH-EQ) for the detected samples (μg DNBP/L).

**Supporting Information, Figure S4.** Observed equivalent (ObTH-EQ) ranges for the source water samples.

**MATERIALS AND METHODS**

***Sampling Preparation.*** Because these water sources are considered to be the safest water body in the contemporary conditions, studies of toxic chemicals in water sources are essential for management and control of priority chemicals in this most developed area in China . No specific permissions were required for the chosen locations/activities and the field study did not involve endangered or protected species.

These waters are considered the most important sources and each provides more than 400 thousand m^3^ drinking water per day. A general population size for each water source is about 500 thousand people.

Composite water samples were collected at each location and placed into brown glass bottles, which were pre-cleaned with nitric acid and chromic acid solution, and then rinsed with high-purity hexane (Merck), dichloromethane (TEDIA), acetone (TEDIA) and methanol (TEDIA). Bottles were also washed 3 times with water samples before sample collection. Samples were transported on ice and stored at 4 °C and extracted within 24 h. Samples were transported and stored at 4 °C and extracted within 24 h. Solid phase extraction (SPE, 500 mg Oasis HLB cartridges, Waters, USA) was performed under vacuum at a flow rate of 6-8 mL/min without filtration. Approximately 2 L of sample was passed through each cartridge to avoid over filtration and was dried completely under a gentle stream of nitrogen gas (>99.9% pure). SPE extracts were combined and dehydrated with anhydrous sodium sulfate and reduced to dryness under a gentle nitrogen flow and reconstituted in 0.1 mL dichloromethane for quantification. Samples used for bioassays were reconstituted in 0.2 mL of dimethyl sulfoxide (DMSO). Extracts were stored at −20 °C. The external standard consisted of Mili-Q water spiked with each target analyte to determine the recoveries of the detected compounds. The internal standards di-n-butyl phthalate-d4, bis(2-ethylhexyl)Phthalate-3,4,5,6-d4 and ^13^C-PCB 141 were added to the tested extracts before instrumental analysis for quality control of phthalate esters, and pesticides. A signal-to-noise ratio of 3 was used as the criteria for the analytical limit of detection (LOD).

Compounds for bioassays were dissolved in dimethylsulfoxide (DMSO, BDH Laboratory Supplies, UK) and then diluted with the appropriate culture medium before use to give less than 0.5% (v/v) solvent.

***MTT Cytotoxicity Test.*** The 3-(4,5-dimethylthiazol-2-yl)-2,5-diphenyltetrazolium bromide (MTT) assay was performed in parallel with the luciferase induction assays, according to the protocol described previously. Briefly, CV-1 cells were plated in 96-well plates using DMEM with 10% dextran-coated charcoal (DCC) serum with the density of 1×10^4^ cells/100 μL. After 24 h incubation, CV-1 cells were treated with vehicle, extracts and standards alone or with 5.0×10^−9^ mol/L T_3_ for 24 h. Then 3-(4,5-dimethylthiazol-2-yl)-2,5- diphenyltetrazolium bromide was added to each well. Absorbance was measured with a microplate reader (EL808, Bio-Tek, Winooski, VT, USA) at 570 nm.

***Reporter Gene Assay.*** Reporter gene assays were conducted according to previous methods with some modification . Cells were plated into 48-well culture plates at a density of 5.0×10^4^ cells per well in phenol red free DMEM medium containing 10% bCDS-FBS. After 12 h, cells were transfected with 0.25 μg Gal4-responsive luciferase reporter pUAS-tkluc, 0.1μg pGal4-L-TR which was an expression vector coding for the ligand binding domain (LBD) of TRβ fused to the DNA binding domain of Gal4, using 2.5 μg Sofast TM transfection reagent per well. After an additional 12 h of incubation, the transfection medium was removed and the cells were exposed to various concentrations of extracts for 24 h. To determine agonistic activity, CV-1 cells were treated with various concentrations of chemicals. For determining antagonistic activity, CV-1 cells were exposed to various concentrations of tested chemicals in the presence of 5×10^-9^ mol/L T3. DMSO concentrations in wells never exceed 0.5% (v/v). The transfection efficiency was 42%. For both agonists and antagonists, luciferase activities of treatment groups were compared to that of the corresponding vehicle control.

***Data Analysis.*** The concentration of standard chemical which caused the same response as the greatest concentration tested (200 times the original concentration) was divided by the enrichment factor 200. In the bioassays, triplicate wells were dosed for each treatment. And the variations between the parallel cells were very small. All the inter-assay standard deviations for the luciferase activities (n-fold of 5 nmol T_3_) were less than 0.02. We repeated the experiments for at least 3 times to make stable and believable results and the related data sets were used for the Monte Carlo simulation.

**RESULTS AND DISCUSSION**

***TR Agonist Activity.*** Phthalate esters exhibited week potencies as agonist.

REFERENCES:

1. Shi W, Zhang F-X, Hu G-J, Hao Y-Q, Zhang X-W, et al. (2012) Thyroid hormone disrupting activities associated with phthalate esters in water sources from Yangtze River Delta. Environ Int 42: 117-123.

2. Sun H, Shen O-X, Wang X-R, Zhou L, Zhen S-q, et al. (2009) Anti-thyroid hormone activity of bisphenol A, tetrabromobisphenol A and tetrachlorobisphenol A in an improved reporter gene assay. Toxicol In Vitro 23: 950-954.
